# Supplementary material for: Essential Roles of Exocyst Complex Component 3-like 2 on Cardiovascular Development in Mice
Source: Life (Basel). 2022 Oct 28;12(11):1730. doi: 10.3390/life12111730 (PMC9694714; doi:10.3390/life12111730)
Supplement: Supplementary file 1 [file life-12-01730-s001.zip › life-1943993-supplementary.pdf]

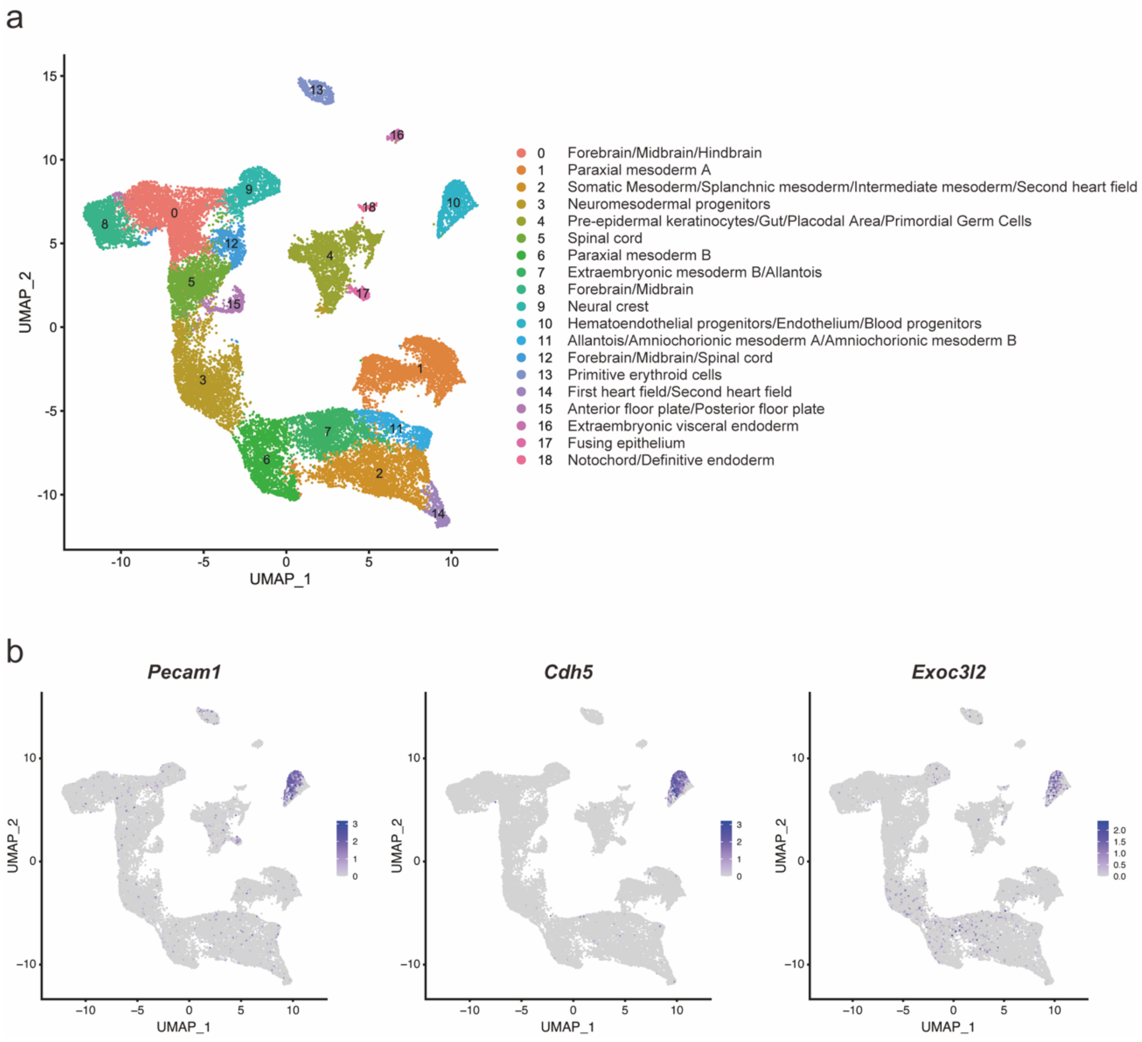

Figure S1.

The expression patten of *Exoc3l2* at E8.5 mouse embryos from sc-RNA-seq data (GSE186069). (**a**) UMAP was generated using a part of E8.5 mouse embryo sc-RNA-seq dataset from GSE186069. Cluster 10 was composed of 51.0% of endothelium. (**b**) The expression levels of endothelial cell markers and *Exoc3l2*. The same UMAPs as in **a**, colored by the expression value of typical endothelial cell markers, *Pecam1* and *Cdh5*, and *Exoc3l2*.

Supplemental Table S1: Primers for mouse genotyping PCR

Strain	Allele	Primer name	Primer seqnce	Amplicon length
Exoc3l2 eGFP KI	WT	161_Exoc3l2_3'arm_Target_Forward	5'- ATAGGATCCTTGTCTTCCCGTCCTCTCCCAACAG -3'	415 bp
		197_Exoc3l2_3'arm_Reverse2	5'- AACCTCCTATCCCAGAAGTCGGTGTC -3'	
	KI	070_GFP-S2	5'- TTCAAGGACGACGGCAACTACAAGAC -3'	517 bp
		197_Exoc3l2_3'arm_Reverse2	5'- AACCTCCTATCCCAGAAGTCGGTGTC -3'	
	Δ	159_Exoc3l2_5'arm_Target_Forward	5'- ATAGGATCCGAAGCCTCTAATGTTTCTAAGAGTGGGACCTG -3'	275 bp
		197_Exoc3l2_3'arm_Reverse2	5'- AACCTCCTATCCCAGAAGTCGGTGTC -3'	
Exoc3l2 flox (*)	WT	218_Exoc3l2_flox_Cent-F	5'- TGCGTCTTGGCTAATTATCTAACTCAGGACC -3'	940 bp (Not digested by EcoRV)
		219_Exoc3l2_flox_3arm-R	5'- TTGCTCTCTCCTTCCACCCTTCCAC -3'	
	flox	218_Exoc3l2_flox_Cent-F	5'- TGCGTCTTGGCTAATTATCTAACTCAGGACC -3'	980 bp (EcoRV produces 334bp + 646 bp fragments)
		219_Exoc3l2_flox_3arm-R	5'- TTGCTCTCTCCTTCCACCCTTCCAC -3'	
	Exon3 Δ	216_Exoc3l2_flox_5arm-F	5'- CTCTCAAACCTATGCCTCCCCTTTCTACAG -3'	875 bp
		219_Exoc3l2_flox_3arm-R	5'- TTGCTCTCTCCTTCCACCCTTCCAC -3'	

* As for Exoc3l2 flox strain, PCR products from WT and flox alleles were distinguished by EcoRV restriction enzyme digestion.

Supplemental Table S2: Primers for qPCR

Target gene	Strand	Primer seqnce
Hprt	Forward	5′- TTGTTGTTGGATATGCCCTTGACTA -3′
	Reverse	5′- AGGCAGATGGCCACAGGACTA -3′
Cdh5	Forward	5′- CCTCTGCATCCTCACCATCACAG -3′
	Reverse	5′- ACCAACTGCTCGTGAATCTCCAG -3′
Pecam1	Forward	5′- GCTGCTCCACTTCTGAACTCCAA -3′
	Reverse	5′- TGGGTTTTACTGCATCATTTCCAGA -3′
Exoc1	Forward	5′- GGAGGAGAACTTACTGCAGGTGG -3′
	Reverse	5′- CGTAACTCCAGATCCAGGGTAGC -3′
Exoc2	Forward	5′- GCATGCAGAGGTATTCACTATTTCC -3′
	Reverse	5′- TTCTGCTGAAGGATGAGACGCAC -3′
Exoc3	Forward	5′- CAGACGTGTGGTGGTGGAATAC -3′
	Reverse	5′- AGCCTCTCTGACCATCTTCTCGG -3′
Exoc3l1	Forward	5′- ATTGAGTTCTGAGTTGCTAGATGGT -3′
	Reverse	5′- CTTAGGTATTGTAACACCACGGTTC -3′
Exoc3l2	Forward	5′- AGATCCTACAGTTGGAGGACACTC -3′
	Reverse	5′- ACCACTTTCAGATAGTTCCAGGTC -3′
Exoc3l3	Forward	5′- CCATTAAGATCGAGGTGGCCACA -3′
	Reverse	5′- ATGCTCCTGACTTCACTGCTTGG -3′
Exoc3l4	Forward	5′- GACACACTTCGTCCCCTCATGG -3′
	Reverse	5′- GTATTCGCGGACCACAAAACGG -3′
Exoc4	Forward	5′- CGGGAGGCAGACTTGGACTTTG -3′
	Reverse	5′- GTACTTCACACCCTGGTCCACC -3′
Exoc5	Forward	5′- CGGGAGAACAACTTGCCAATCTG -3′
	Reverse	5′- TGCCTTTGTAAATTCAGCTCAGC -3′
Exoc6	Forward	5′- ACATATTCGCCCAGTTCCGGAAG -3′
	Reverse	5′- ACATACCAGTCACCAAGCCTCTG -3′
Exoc6b	Forward	5′- GAACCCTGTGACAGCTCTGACC -3′
	Reverse	5′- GCCACGGTGTCAATCAATTTCTGC -3′
Exoc7	Forward	5′- GCGGCAGATGATTAAGGAACGTTTC -3′
	Reverse	5′- TCTCTGCTCTGTGTCTGGAATAGC -3′
Exoc8	Forward	5′- AGGAGATGAAAAGTTGCGGGGTC -3′
	Reverse	5′- CCCATAGTCTGCTTGGTGAAGGC -3′
